# Supplementary material for: Culex pipiens and Culex restuans egg rafts harbor diverse bacterial communities compared to their midgut tissues
Source: Parasit Vectors. 2020 Oct 27;13:532. doi: 10.1186/s13071-020-04408-4 (PMC7590256; doi:10.1186/s13071-020-04408-4)
Supplement: Supplementary file 1 — Additional file 1: Table S1. SIMPER analysis of the major bacterial OTUs driving differences between sample treatments (mosquito species and life stage). The Bray-Curtis average dissimilarity between sample treatments was >1% for 18 bacterial taxa. Overall average dissimilarity. CXP.EG – Cx. pipiens egg raft samples; CXP.MG – Cx. pipiens midgut samples; CXR.EG – Cx. restuans egg raft samples; CXR.MG – Cx. restuans midgut samples. Figure S1. Rarefaction curve analysis of observed richness of bacterial OTUs of samples from Cx. pipiens and Cx. restuans midgut and egg samples. Figure S2. Venn diagrams showing the number of unique and shared bacterial OTUs between egg and midgut samples of Cx. restuans and Cx. pipiens. a Venn analysis of bacterial OTUs from all four sample types. b Venn analysis of bacterial OTUs from Cx. pipiens and Cx. restuans midgut samples. c Venn analysis of bacterial OTUs from Cx. pipiens and Cx. restuans egg samples. d Venn analysis of bacterial OTUs from Cx. pipiens eggs and midguts. e Venn analysis of Cx. restuans egg and midgut samples. [file 13071_2020_4408_MOESM1_ESM.docx]

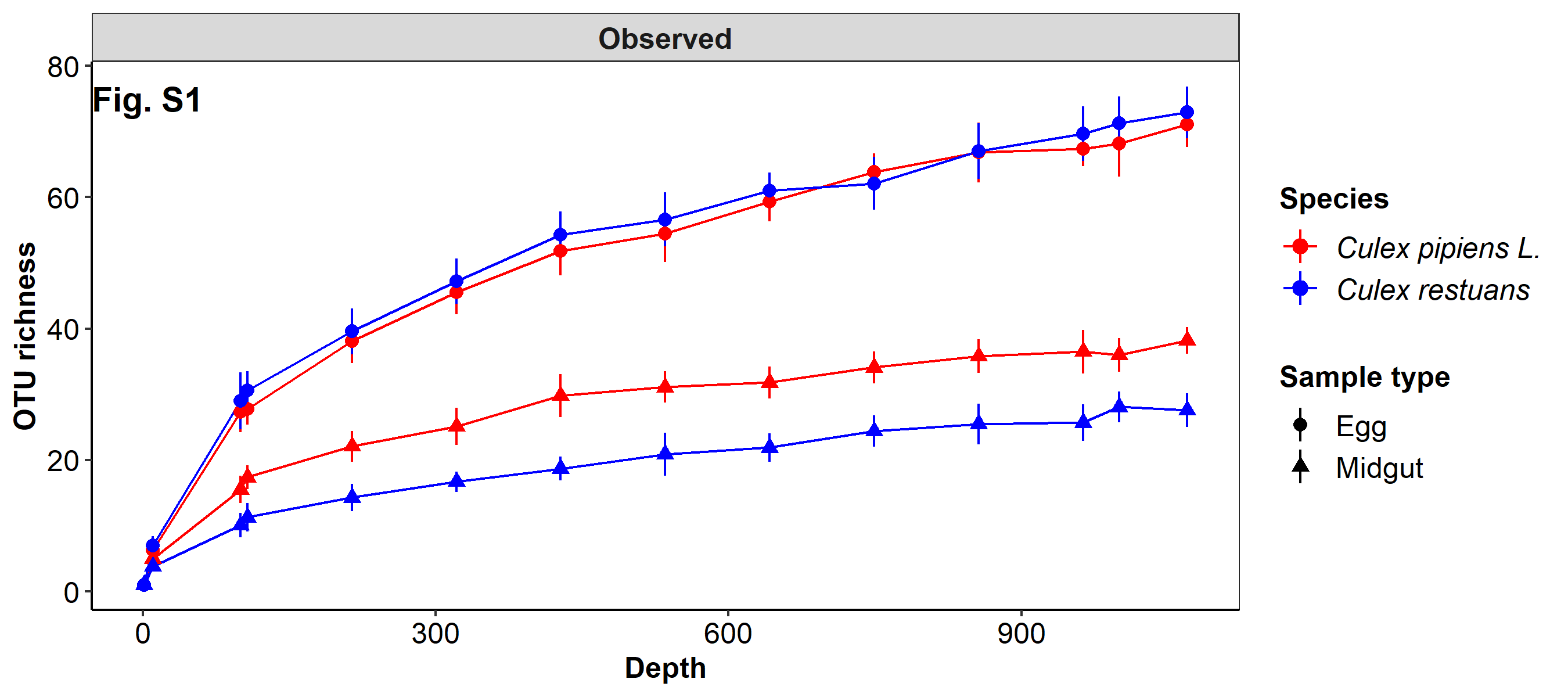


**Additional file 1: Figure S1** Rarefaction curve analysis of observed richness of bacterial OTUs of samples from Cx. pipiens and Cx. restuans midgut and egg samples


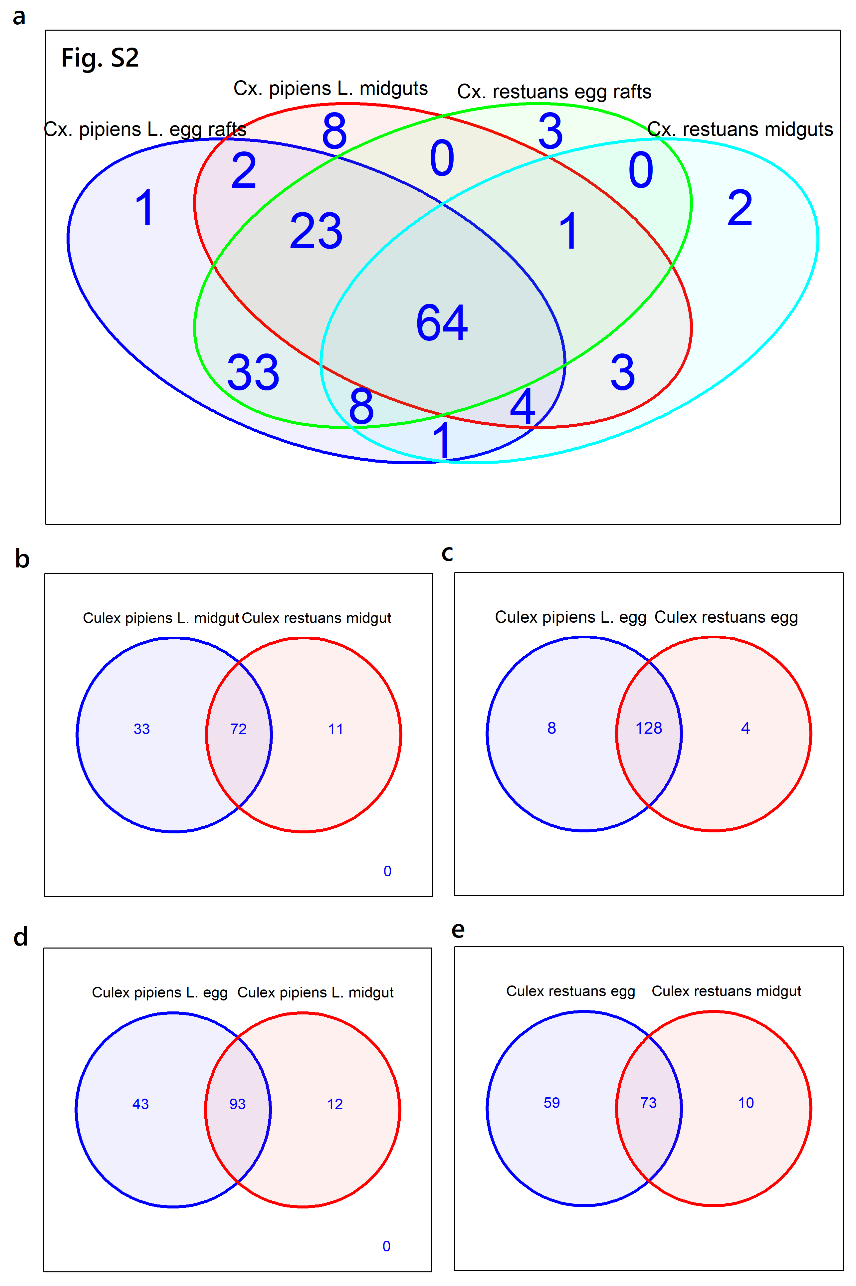


**Additional file 1: Figure S2.** Venn diagrams showing the number of unique and shared bacterial OTUs between egg and midgut samples of Cx. restuans and Cx. pipiens. a: venn analysis of bacterial OTUs from all four sample types; b: venn analysis of bacterial OTUs from Cx. pipiens and Cx. restuans midgut samples; c: venn analysis of bacterial OTUs from Cx. pipiens and Cx. restuans egg samples; d: venn analysis of bacterial OTUs from Cx. pipiens eggs and midguts; E: venn analysis of Cx. restuans egg and midgut samples

**Additional file 1: Table S1**. SIMPER analysis of the major bacterial OTUs driving differences between sample treatments (mosquito species and life stage). The Bray-Curtis average dissimilarity between sample treatments was >1% for 18 bacterial taxa. Overall average dissimilarity. CXP.EG – Cx. pipiens egg raft samples; CXP.MG – Cx. pipiens midgut samples; CXR.EG – Cx. restuans egg raft samples; CXR.MG – Cx. restuans midgut samples.

| **Taxon** | **Average dissimilarity** | **Percent contribution** | **Cumulative percentage** | **CXP.EG** | **CXP.MG** | **CXR.EG** | **CXR.MG** |
| --- | --- | --- | --- | --- | --- | --- | --- |
| *_Providencia_* | _17.11_ | _19.14_ | _19.14_ | _4_ | _4630_ | _7_ | _6060_ |
| *_Ralstonia_* | _9.453_ | _10.57_ | _29.71_ | _2010_ | _125_ | _1490_ | _47_ |
| *_Novosphingobium_* | _9.247_ | _10.34_ | _40.06_ | _1250_ | _900_ | _1880_ | _153_ |
| *_Spironema_* | _7.497_ | _8.386_ | _48.44_ | _6_ | _217_ | _79_ | _7000_ |
| *_Wolbachia_* | _7.454_ | _8.338_ | _56.78_ | _415_ | _2430_ | _0_ | _7_ |
| *_Acinetobacter_* | _4.563_ | _5.104_ | _61.88_ | _683_ | _118_ | _624_ | _565_ |
| _Methylobacteriaceae_ | _2.957_ | _3.307_ | _65.19_ | _163_ | _23_ | _1040_ | _19_ |
| *_Methylobacterium mesophilicum_* | _2.58_ | _2.886_ | _68.08_ | _478_ | _19_ | _371_ | _1_ |
| *_Flectobacillus_* | _2.503_ | _2.799_ | _70.88_ | _288_ | _152_ | _533_ | _9_ |
| _Enterobacteriaceae_ | _2.319_ | _2.593_ | _73.47_ | _28_ | _460_ | _174_ | _274_ |
| *_Erwinia_* | _2.163_ | _2.42_ | _75.89_ | _0_ | _109_ | _0_ | _871_ |
| *_Ramlibacter_* | _2.084_ | _2.331_ | _78.22_ | _293_ | _1_ | _399_ | _1_ |
| _Comamonadaceae_ | _1.551_ | _1.735_ | _79.95_ | _283_ | _49_ | _247_ | _3_ |
| *_Aquitalea_* | _1.337_ | _1.496_ | _81.45_ | _184_ | _40_ | _285_ | _0_ |
| *_Sphingomonas yabuuchiae_* | _1.22_ | _1.365_ | _82.81_ | _239_ | _3_ | _136_ | _2_ |
| *_Serratia_* | _1.075_ | _1.202_ | _84.02_ | _39_ | _218_ | _0_ | _24_ |
| *_Novosphingobium capsulatum_* | _1.031_ | _1.153_ | _85.17_ | _176_ | _28_ | _191_ | _8_ |
| _Methylophilaceae_ | _1.016_ | _1.137_ | _86.31_ | _170_ | _0_ | _167_ | _1_ |
